# Epilepsy & behavior reports: Editorial of the immediate past editor-in-chief

**DOI:** 10.1016/j.ebr.2023.100594

**Published:** 2023-02-24

**Authors:** William O. Tatum

**Affiliations:** IV, DO FAAN, FACNS, FAES Immediate Past Editor-in-Chief, Epilepsy & Behavior Reports Professor of Neurology, Mayo Clinic College of Medicine & Health Sciences Jacksonville, Florida USA

As the pandemic wanes and 2023 has begun, Epilepsy & Behavior Reports (EBR) has many people to thank for publishing a number of high-quality submissions during the course of the pandemic. Our goal at EBR is to become the most-read epilepsy journal available on the world-wide web for the public via open access. Novel case reports and case series, full-length manuscripts, review articles, short communications, and letters to the editor are acceptable formats. Lay abstracts, topical issues, special issues and more in 2022 further enhanced the journal by increasing the use of social media. We hoped to increase awareness of our relevant articles to help clinicians manage their patients with epilepsy and the behavioral consequences. By highlighting clinical epilepsy, the surgical approaches to treatment, new information on antiseizure medication, pertinent semiology and behavior, scalp/intracranial EEG, psychological and social issues, we hope to add to new approaches facilitating tools to guide accurate diagnosis and personalized treatment to patients with epilepsy.

In the last year EBR has grown with our editors and editorial board to boast many achievements. Our peer-reviewed journal engages an average of at least 2 reviews/submission. Many thanks to Steve Schachter as founding editor-in-chief for the opportunity to work alongside many wonderful colleagues with a singular focus. The experience has been unforgettable and an adventure. Thank you to Jerzy P. Szaflarski MD, PhD for assuming the next role as editor-in-chief. On 12/31/2022 I completed a 7 year path with the comforting thought that EBR would be meticulously cared for by my successor. Many thanks to Dr. Kurupath Radhakrishnan for an amazingly strong partnership as Associate Editor. Equally so, to Dr. Barry Gidal for providing longitudinal and outstanding input and article summaries for our lay readership. Thanks to Dr. Arthur Grant for taking on an impossible role with vigor, and Dr. Sallie Baxendale for her concept of inaugural Special Issues. To the entire Editorial Board including past Associate Editors, Drs. Joe Sirven and Dorothee Kasteleijn-Nolste, thank you for your constant support! I have always appreciated the advice and support you have graciously given me over the years. Special thanks to Mehroon Farooqui who works tirelessly to make the entire EBR engine run. You have all been a delight to work with managing EBR. For our reviewers who dedicate their expert volunteer time providing timely reviews- my sincere gratitude and appreciation.

To produce a top-ranked journal, there are multiple members of the team required. We welcome Mary Ma as our newly appointed publisher, and do not forget Daniel Staemmler and Annie Zhao for their past personal support to the journal. As the first quarter of 2023 unfolds, we look forward to acquiring an impact factor for EBR this year. Many new and exciting ideas are evolving under new leadership, and as I close my tenure at EBR, I am humbled by the confidence given to me by Dr. Schachter, past and present associate editors, the EBR board, expert reviewers, and our colleagues at Elsevier who helped make my work as editor-in-chief successful to bring novel and relevant publication to our readers. If you are considering sharing or learning from key reports on rare, unexpected, and unique discoveries in people with epilepsy and the behavioral consequences, EBR is right for you.

